# Medical Items and News of Interest to the Physician

**Published:** 1874-04

**Authors:** 


### For the Use of Physicians.

CULLED FROM THE STANDARD MEDICAL JOURNALS OF THE WORLD.

NOTE.—These formulæ are intended only for the regular practitioner of medicine, and should be employed only by him, or under his immediate supervision.

**Burns.**

At Rosevelt Hospital they use—ol. linim. et aquæ calcis, āā.

**Epithelioma of Eye.**

A patient with this disease experienced great relief from pain by the application of citric acid in moderately strong solution. The value of this remedy, for this purpose, has been known for some time.

**Adhesive Plaster.**

According to Otto Facilides, adhesive plaster, which has become brittle by age, and has lost its adhesive qualities, may be rendered adhesive again by coating it with turpentine, by means of a sponge, and leaving it exposed for a day.

**Treatment of Chronic Eczema of the Genitals.**

Dr. de Montmya recommends the use of tincture of iodine in the treatment of the chronic eczema and intertrigo of the genitals, more especially combined with the use of a lotion of one part of corrosive sublimate to 250 of water, a few drops of spirit being used to dissolve the corrosive sublimate.

**Mixture for Acute Articular Rheumatism.**

The alkaline treatment is the one most generally adopted in the medical wards of Bellevue Hospital, New York. A prescription which has found great favor is as follows:

℞
Sodæ bicarb., ℥ iss.
Potass. acetat, ℥ ss.
Liq. ammon. acetat, f ℥ iij.
Aquæ, q. s. ad., Oj.—M.
℞ Acid. citric, ℥ ij.
Aquæ, Oi.—M.
℥ ij. of each q. 3 vel 4 h.

This forms an agreeable effervescing mixture, rendering the urine alkaline very quickly. The frequency with which it is given depends upon the degree of alkalinity obtained as tested morning and evening.—Dr. FARRINGTON in *Phila. Med. Times.*

**Irregular Action of the Heart**

is very nicely controlled in very many cases by the administration of ext. nuxvomica, gr. ¼, three times a day.—*Bellevue Hospital Reports—Record.*

**Hydrocele.**

A somewhat remarkable result was obtained in the treatment of a case of hydrocele by using an injection of pure Lugol's solution. The entire tunic vaginalis was removed, and the patient was making a rapid and satisfactory recovery.—*Ibid.*

**Administration of Ether.**

The administration of potass. bromid, grs. xxv., previous, and the same amount immediately following, or as soon as the patient can conveniently swallow, after the administration of sulphuric ether for the purpose of producing anæsthesia, is now regularly resorted to. The effect is to prevent the vomiting which so commonly follows the use of the anæsthetic.—*Ibid.*

**Chronic Synovitis.**

This class of cases are now being treated by applying passive motion, as much as is possible for the patient to bear without producing pain. If pain is developed, place the patient in bed, secure perfect rest, and as soon as the inflammation has subsided, again resort to passave motion. It is feared some kind-hearted doctor will get into difficulty in attempting to apply this principle in private practice.—*Ibid.*

**Cough Mixture in Consumption.**

℞
*Mist. Glycyrrhizæ Comp.*, ℥ iv.
Syrup. pruni virg., ℥ ij.
Quininæ, gr. xxx ij.
Morphiæ sulphat., gr. ij.
Elix. taraxaci comp., ℥ ij.
Mix.—Dessertspoonful every four hours.

**Remedy for Chronic Hoarseness.**

An eminent physician of Philadelphia contributes the following: In chronic hoarseness arising from thickening of the vocal chords and adjacent membrane, the ammoniated tincture of guaicum is often a very effiacious remedy. It may be appropriately mixed with equal parts of the syrup of senega, and a teaspoonful of the mixture given two or three times a day.

**Erysipelas.**

The good old-fashioned liq. plumbi et opii is relied upon as an external application. Internally, tr. ferri muriatis is administered regularly.—*Rosevelt Hospital Reports.*

---

**Night Sweats in Phthisis.**

When sulphuric acid, dilute or aromatic, has failed to give relief in these cases, the following is given every night in the form of pill:

℞
Zinci oxid, grs. ij.
Ext. hyoscyami, grs. iij.—M.

For the diarrhœa of phthisis, persulphate of iron or injections of tr. opium and starch are used.—*Ibid.*

---

**Pneumonia.**

The plan of treatment generally adopted in the treatment of this disease consists in administering quinine in six-grain doses night and morning, with plenty of stimulants and plenty of nourishment in the form of milk and beef tea. All the cases which have been admitted to the hospital have received this treatment, and almost all have done exceedingly well.—*Ibid.*

---

**Morbus Brightii.**

The chief elements entering into the treatment of this disease are tonics, diuretics, and diaphoretics. It is quite commonly the case that the patient is not seen by the physician until some dropsical symptoms have made their appearance, such as puffiness about the eyes, or slight swellings about the ankles. It is the "dropsy" that has alarmed the patient. In such cases quinine and tr. chloride of iron, associated with infusion of digitalis in combination with bitartrate or ascitrate or citrate of potassa, constitute the remedial measures ordinarily employed. If there is much œdema, the hot-air bath is called in to assist, and occasional doses of the following pill is given:

℞
Pil. hydrarg.,
Res. scammon.,
Pulv. aloes, āā grs. x, M.

and divide into ten pills.—*Ibid.*

---

**Nocturnal Pains.**

A majority of the cases of nocturnal pains can be rid of their remorseful reminders by the administration of iodide of potassium in free doses; but occasionally one presents itself in which either the demon's fang has penetrated more deeply, or the patient is more susceptible to grievances, and these "nocturnal aches" will scarcely yield to the single-handed onset of iodide of potassium. In these rebellious cases, Dr. Drake, now in charge of the venerial service, administers, with the happiest results, a combination of three iodides, as follows:

℞

Pot. iodid., ʒ iv.;

Sodæ iodid., ʒ iij.;

Ammonium iodid., ʒ ij.;

Tr. gentian co. ad. ℥ ij.—M.

Sig.:—ʒ j. t. i. d.—*Record.*

---

**Improved Tests for Saccharine Urine.**

Dr. Seegen's modification of Trommer's test consists in the preliminary filtration of urine through animal charcoal. This removes the uric acid and other matters which interfere with the action of Trommer's test, and leaves a colorless fluid, which is highly sensitive to the test. It is a delicate qualitative, but not a quantitative method.—*British Med. Journal.*

---

**Angina Pectoris.**

This is one of those neuralgic affections which fills the patient with fearful apprehensions, in addition to the actual suffering experienced. The physicians of Rosevelt Hospital, New York, administer quinine and iron regularly, while the attacks of angina are subdued by Hoffman's anodyne, in drachm doses, p. r. n.

---

**Oleate of Mercury in Treatment of Ringworm.**

Dr. Kane (*London Lancet*) describes several cases in which he had used the oleate of mercury in treating ringworm. He says that over other remedies it has the following advantages: (1) It is a *certain remedy* if carefully applied. (2) It *produces no staining* or injury of the skin. This is of great importance where the disease appears on the face. (3) It is *painless* in its application, which is not true of the strong parasiticides. (4) It *readily penetrates* the sebaceous glands, hair-follicles, and even into hairs themselves. Thus it is more likely to destroy the fungus than spirituous or aqueous solutions of mercury.

In very sensitive skins the irritation sometimes produced by it may be avoided by reducing the strength of the solution, and applying it with a camel's-hair brush. The ordinary strength of the oleate is ten per cent.

**Aromatic Candles.**

The latest novelty in the way of pharmaceutical applications, is a candle impregnated with some aromatic substances, such as benzoic acid, or styrax. When the candle is lighted, the surrounding air is quickly impregnated with balsamic vapors, which, it is thought, will afford relief to many cases of irritation of the pulmonary mucous membrane, as well as to bronchial spasm.—*Boston Med. and Sur. Journal.*

---

**Subcutaneous Injection in 1664.**

It seems to be again demonstrated that there is "nothing new under the sun," as will more clearly appear from the following extract taken from *Pepys's Diary*, vol. iii, p. 126 :—

"Went with Mr. Pierce, the Surgeon, to see an experiment of killing a dog, by letting opium into his hind leg. He and Dr. Clarke did fail mightily in hitting the vein, and in effect did not do the business after many trials ; but with the little they got in, the dog did presently fall asleep, and so lay till we cut him up."

---

**Emulsion of Pancreatine, Pepsin, and Cod-Liver Oil Compound.**

℞

Olei morrhuæ (Moller's) ℥xij.

Pepsinæ (Sheffer's ℞) ʒ iiss.

Pancreatinæ (Mosron's) ʒ iiiss.

Tinct. phosph. (homœop. mother tinct) ʒ ss.

Olei cinnamomi veræ, gtt. xxxij.

Olei amygdalæ amaræ, gtt. xvj.

Glycerinæ (C. P.) ℥ iv.

Sol. acidi carbolici, No. 4, gtt. xvj.

Mix.— Sig.— Take a tablespoonful three times a day before meals.—*Druggist's Circular.*

---

**Strumous Children.**

This class of cases embraces those children who seem to be favored with eczematous eruptions, conjunctivitis, glandular enlargements, and exhibit in general a predisposition to inflammatory affections, especially of the mucus membranes.

The following prescription has been found to be of great service in the therapeutical management of these patients :

℞

Syrup pyrophosphate of iron.
Syrp. lacto-phosphate of lime, āā f. ℥ iv.
M.

Sig :—A teaspoonful three times a day.—*Record.*

**Peri and Endo-Carditis and Acute Articular Rheumatism.**

The treatment adopted in Bellevue Hospital is as follows :

℞

Potass. acetat., ℥ ss.;
Liq. ammon. acetatis, ℥ iij.;
Sodæ bicarb., ℥ j.—M.

℞

Acid citricum, ℥ ij.;
Aquæ, O. j.—M.

These two mixtures are to be consumed in twenty-four hours, as effervescing draughts.

For an anodyne mixture the following is given :

℞

Sol. morph., (Mg.) ʒ iss.;

Fld. ext. bell., ʒ ss.;

Aqua cinnamomi, ℥ ij.—M.

Teaspoonful p. r. n.—*Medical Record.*

---

**Caoutchouc Electuary as a Remedial Agent.**

Dr. Theodore Varick, after having for fifteen years prescribed caoutchouc in preference to cod-liver oil in certain cases of pulmonary tuberculosis in every stage, in chronic bronchitis, the winter coughs of old people, and in chronic rheumatism, desires to call attention to its use as a remedial agent. It is not claimed that it is a remedy which has any specific action, but that it diminishes excessive mucous secretion and suppuration, arrests hemorrhage and colliquative sweating, and retards emaciation.

Prepared in the following manner, it should be given in doses of a teaspoonful three times a day, about two hours after meals :

℞

Caoutchouc (in thin slices,) ℥ i.

Olei terebinthinæ, f ℥ ii.

Mix.

℞

Sol. caoutchouc, f ʒ ij.

Sacch. alb., f ℥ iss.

Mellis (strained) f ℥ iiss.

Mix.

When these preparations are mingled, the mixture (containing about two grains of caoutchouc to the teaspoonful) should be of an opaque yellow color and thick enough to run very slowly from a spoon. One hundred parts of caoutchouc contain 87.2 carbon and 12.8 hydrogen, while cod-liver oil contains of carbon 37 and hydrogen 34— ; so that should the former be available as respiratory force or fuel it would be of great value.—*New York Medical Journal.*

**Neuralgia in Infants.**

Children from two to six weeks old, especially males, suffer frequently with attacks of pain in the bowels, coming on about midnight, and lasting until four or five in the morning. Children thus affected cry violently, but towards morning become quiet, fall asleep, and the next day are as well as ever. This enteralgia does not seem to be caused by any fæcal accumulations; it is very noticeable, however, that during the paroxysm they pass no water, and at the end of it a large quantity of pale colored urine comes away, as after an hysterical attack. The cause of this retention of urine is unknown. The disease affects children of all classes of society, indiscriminately, without reference to their hygenic condition. The remedy recommended by Dr. Boyd (*Edinburgh Medical Journal*, Feb., 1873,) is spiritus ætheris nitrosi, eight or ten drops in a drachm of water. Immediately afterwards, with an escape of wind, the crying ceases, and the little patient goes to sleep.

---

**Ergot in Epistaxis.**

Dr. Andrew H. Smith (*The Medical Record*, October 15, 1873,) reports the case of a man, æt. 37, who suffered from persistent epistaxis, recurring sometimes two or three times a day. Direct and rhinoscopic examinations showed no abnormal condition of the nasal mucous membrane. Astringents were applied locally by means of both the brush and syringe, and such general treatment was resorted to as the symptoms demanded. After this had been persevered in for two weeks without affecting the hemorrhages, the fluid extract of ergot was prescribed in twenty-drop doses three times a day.

This was continued for ten days, with the effect of entirely restraining the bleeding from the time the first dose was taken. The medicine was then omitted, but in a few days the bleeding began anew. It was immediately arrested by a resort to the drug, and did not afterwards return; the medicine being continued at gradually increasing intervals for nearly a month.

---

**Treatment of Erysipelas.**

Dr. Kraczorowski, in a Berlin medical journal, reports a new mode of treating erysipelas, since the introduction of which into several of the hospitals at Posen no fatal case has occurred. The affected parts of the skin are gently painted over every three hours with a mixture of one part of carbolic acid and three parts of oil of turpentine; and the same is rubbed somewhat more energetically into the adjacent parts. Compresses dipped in lead ointment are then applied, and lastly, according to the extent of the disease, either an ice-bag is laid upon the part, or the whole limb is wrapped around with the cloths dipped in ice-cold water; and these must be promptly changed for fresh ones as soon as they become at all warm. When adynamic symptoms are present, Hungarian wine is given, and camphor mixture. The bowels should be kept open. The parts rubbed with the mixture become very red and even blistered; but the epidermis soon dries and the skin appears as if tanned. The progress of the disease is arrested in from twenty-four to thirty-six hours, and no relapse occurs.

---

**Magnesia as a Surgical Dressing.**

Dr. Ohlmeyer, of Weissenburg, has found the carbonate of magnesia of value:

1. In atonic ulcers.

2. In cases where the epidermis was eroded and the subjacent tissues were the seat of pain, and were prone to subsequent suppuration.

3. In relieving the pain of inflamed wounds.

4. In cases where it was desirable to stimulate the affected surface, prevent the access of air, and limit the formation of pus. He was led in the first instance to try this remedy from its well-known action in those states of the stomach where there is an excessive formation of acids. These latter, uniting with the base magnesia, are neutralized, and carbonic acid evolved. Accordingly, he believes that in exposed surfaces where the process of healing is prevented by fermentative action, this dressing is indicated. The use of it was attended with satisfactory results. The magnesia unites with the acids which form on the surface; it excludes the oxygen, forms an artificial covering, irritates the granulations, and forms a barrier against external and harmful agents.

In preparing the application he selects a fluid that will not readily oxidize. Oil answers this indication, and the kind he employs is the oil of sweet almonds. Adding to this the carbonate, he makes a tolerably fluid paste or salve. This is then spread upon linen and laid over the wound. It is held in place in the ordinary way.

Dr. Ohlmeyer also adds that he has used the carbonate successfully in facial erysipelas, when it was important to protect other patients from infection. In the latter case he used water as a substitute for oil.—*The Clinic.*

---

**Pneumatic Aspiration in Hydrarthrosis of the Knee.**

Dr. Rasmussen, of Copenhagen, describes seven cases in which he practiced aspiration in eight knee-joints. In none of these cases did the slightest trace of inflammatory re-action follow the operation. Even in the cases where there had been severe pains and considerable tenderness of the joint, it was so free from both for several days after the operation that treatment by *massage* could be employed for the removal of the remainder of the effusion. He advises the operation in both the chronic and acute forms of this affection. The following is Dr. Rasmussen's mode of operating : Broad strips of adhesive plaster, clipped at the ends, are applied above and below the joint, and are then gradually tightened according as the evacuation of the fluid by suction, which takes place very slowly, proceeds. By the continued application of adhesive plaster the fluid is forced towards the canula. This should, as a rule, have a diameter from .08 to .06 of an inch, so that the viscid portion of the fluid can pass through it. At first, Dr. Rasmussen made the puncture through the extensor muscles, in the pouch of the capsule, for fear that the fluid would continue to ooze through the comparatively large opening, if the latter were to be made at a lower level. This subsequently, however, was found to be both unnecessary and inexpedient, for by this plan the fluid could with difficulty be drawn off, and the puncture is now made in an upward direction at the external edge of the patella. After the removal of all of the fluid, the opening is closed with charpie and collodion, and the last strip of plaster is applied in the centre, and thus the joint is compressed and placed at rest. To secure rest more completely, the leg is bandaged and an ice bladder is applied to the knee, although the latter is thought to be, perhaps, superfluous.

Occasionally slight œdema of the foot and ankle follow the operation ; but clipping of the plasters, and so loosening them, causes it to disappear. The bandage may be removed at the end of three or four days, and the fluid will commonly have disappeared. When it it has recurred it is in less amount, and the joint is quite free from pain. The utmost care is essential in disinfecting the trocar, and in operating in a locality where there is neither pyæmia nor erysipelas.—*Record.*

---

**Nitrite of Amyl in Angini Pectoris.**

Having recently experienced in his own person (says Dr. W. Herries Madden in the London *Practitioner*,) the remarkable beneficial action of the nitrate of amyl in cutting off short attacks of angina pectoris, Dr. Madden records what he felt. After a sharp attack he at once inhaled five drops, and the effect, he says, was truly wonderful. The spasm was, as it were, strangled at its birth. It certainly did not last two minutes, instead of twenty, as formerly. And so it continued. The frequency of the paroxysms was not diminished for some time ; but then they were mere bagatelle as compared with their predecessors, and consequently the drain upon the vital energies was greatly reduced. Under these improved circumstances strength gradually returned ; the attacks became less and less frequent, and finally ceased. Dr. Madden does not profess to give a full scientific description of the phenomena presented by the nitrate of amyl in action. The first effect was often bronchial irritation causing cough ; then quickened circulation; then a sense of great fullness in the temples, and burning of the ears; then a violent commotion in the chest, tumultuous action of the heart, and quick respiration. The angina pain then died out, first in the chest, next in the left upper arm, and last of all in the wrist, where it was usually extremely severe. There was not at any time the slightest confusion of thought, or disturbance ot vision, but occasionally slight and transient headache. As regards physical signs, the rasping sound was soon modified; but a loud blowing systolic murmur —heard at the base of the heart, along the aorta and in the subclavians, especially the right—continued throughout the illness. One curious feeling which Dr. Madden commonly had was that the front of the chest seemed to be bulged out in a convex prominence, which suddenly terminated at the lower end of the sternum in a sharp and deep depression towards the spine. This was a purely subjective phenomenon. There was no contraction of the diaphragm, nor retraction of the abdominal walls.

In *slight* commencing attacks merely smelling the cotton-wood on which a previous dose had been poured was sufficient to relieve the pain. It acted like a gentle anæsthetic, without any quickening of the circulation. But in a *severe* attack the full action of the drug, with its concomitant vascular commotion, was quite essential. The pain never began to yield until the heart was violently affected.

---

**On the Arrest of Destruction of the Lung in Chronic Phthisis by the Inhalation of the Vapors of Oxygenated Essences.**

"1. The vapors of oxygenated essences, like the powders of these substances, have the property of arresting the destructive progress of phagedænism, and of favoring the reparation of obstinate ulcers of the cornea, etc. Nothing similar is to be obtained from non-oxygenated essences, of which the essence of turpentine is the type

"2. The pulmonary caverns of phthisis, when treated by inhalation of these vapors, undergo cicatrization under the influence of this mode of treatment in a great number of cases and in a relatively short period of time.

"3. The expectorated material of all the patients treated in this manner was examined according to Fenwick's method. The presence of the elastic fibres, made out at the commencement, and their disappearance, made out at the end of the treatment, justify the employment of inhalation of oxygenated essences in vapor during the ulcerative period of chronic phthisis.

"4. The free vapors of these substances have but a very feeble tension, and consequently do not sufficiently charge the atmosphere of apartments in which they are allowed to spread. I have, therefore, in my attempts made use of a small vaporizing apparatus, constructed by M. Collin according to my instructions. This drives the air saturated with the vapor into the bronchial tubes with more force.

"5. All the oxygenated essences may be employed with the chance of success. I have made use of the oxygenated essence of Japanese camphor, of cammomile, of cedar, of eucalyptus or eucalyptol.

"6. I prefer the oxygenated essence of *laurus camporæ*, the odor of which is less penetrating than that of the Borneo camphor. The oxygenated essence of cedar, the odor of which is agreeable and mild, is very well tolerated by patients.

"7. Intense continued fever, great debility, rapidity in the course of the disease, emaciation, all represent so many conditions unfavorable to the success of these inhalations.

"8. The mild and torpid form of phthisis, with partial conservation of strength, abundant expectoration, cough, and oppression, present conditions under which the inhalation of vapors of oxygenated essences give the best results.

"9. Under the influence of this mode of treatment the expectoration, cough, and dyspnœa are relieved, the appetite restored, the strength increased, the hectic fever reduced, and the weight of the patient raised. Finally, in a very great number of cases I have had the satisfaction of observing a disappearance of all the morbid phenomena and a restoration of the patient to good health.

"10. The employment of this means should not in any way contraindicate the use of other medicinal agents, and of the usual regime, for it has no specific pretension. It averts the pulmonary destruction, and allows the physician to utilize this remission to the advantage of the patient in combating by appropriate means the tuberculous diathesis.

"11. Here we have but the application of a property of oxygenated essence which has been well determined in recent times. One cannot see any point of contact between this with the unfortunate suggestions which have led some to regard camphor as a panacea, and have at the same time set many physicians against the truly rational employment of this therapeutical agent."—Dr. Cherom in *Gazette Hebdomadaire*.

---

**Mint for the Suppression of Milk.**

Dr. Dasara observes that the knowledge of the antilactiferous properties of mint appears to have been possessed in very ancient times, since Dioscorides mentions the fact in his works, and subsequent writers have only confirmed his statement. Linnæus observed that cows that ate mint in their pastures yielded a very serous milk, and Laewis affirmed that the coagulation of milk in which some leaves of mint were placed was retarded. More recently M. Desbois de Rochefort, experimenting on mint, found that fomentations of mint applied to the breast, and the infusion taken internally, were capable of suppressing the lacteal secretion, and of preventing the usual accidents attending milk fever in puerperal women. Trousseau expressed some doubt respecting this action of mint, in his treatise on Materia Medica ; but Dr. Pasquale Pepre, in a note on Trousseau's observation, remarks that the fresh leaves of mint placed in the axilla are commonly used in Naples to suppress the milk. Dr. Dasara determined to experiment for himself, and gives the details of a series of cases in which he tried the effects of the application of mint poultices made from the young sprigs at various periods of lactation, and the following are the conclusions at which he has arrived : 1. It is an established fact that mint has the power of suppressing the lacteal secretion ; 2. The suppression of the secretion takes place at whatever period of lactation the mint is employed ; 3. The effect takes place in a very short space of time, according to his experiments in from three to five days ; 4. The suppressive action of mint can be localized to one breast ; 5. No danger nor even any inconvenience arises either to the mother or child, either from the use of the mint or from the suppression of the secretion.—*Rivista Teorico Practica.*

---

**Atropia in Phthisical Sweating.**

A notice in the *Philadelphia Medical Times* of last year, in which Dr. J. C. Wilson stated that he had successfully used sulphate of atrophia in doses of one-eightieth of a grain for the relief of profuse sweating in four cases of phthisis, led Dr. Frantzel to make an extended series of researches in the Charite Hospital in Berlin on the effect of atropia in such cases. In a paper on the subject published in *Virchow's Archiv*, vol. lxviii., part i. (*Allegemeine Medicin, Central Zeitung*, August 2,) he states that, having given it to seventy-five cases, he has arrived at the conclusion that it is a remedy that he can confidently recommend not only in phthisical sweating, but also in that which attends other diseased conditions, such as acute articular rheumatism and convalescence from trichinosis. Among the seventy-five cases were fifteen cases of more or less recent cheesy pneumonia, of whom all had more or less fever with night sweats ; forty-eight of distinct pulmonary phthisis, of whom forty-two had hectic ; one of acute articular rheumatism with high fever; two of ulcerative endocarditis, and two of trichinosis. In the first fifteen patients the sweating was in six completely arrested, in seven much diminished, in two there was no change. In the forty-eight phthisical cases, the medicine had no effect in five, in twenty-one the sweating was remarkably abated, and in twenty-two it disappeared entirely. Several of the patients in whom the atropia failed were near death when it was given. In the eight cases of rheumatism the atropia gave permanent relief in five, in two it produced a marked diminution of the sweats, in one it was useless. In one of the cases of ulcerative endocarditis it proved useful ; not so in the other. In the two cases of trichinosis the cessation of the acute stage of the disease, and of the hectic fever attending it, was followed without any rise of temperature by profuse night-sweats. Sulphate of atrophia, in doses of a milligramme (.015 grain,) was given two hours before the expected access of sweating daily for five days in succession in one case, and for three days in the other ; the result being that the sweats entirely disappeared from the first evening when it was given. In one of the cases of rheumatism, in a man aged thirty-two, nearly all the large joints of the upper and lower limbs had been severely affected during five days; the patient was covered with sudamina, and, when seen by Dr. Frantzel, was bathed in sweat. A milligramme of sulphate of atropia, was given immediately, and very soon there was an abatement of the sweating, which in two hours disappeared. It returned in the night, but ceased the next forenoon after the administration of a similar dose. The atropia was thenceforth given regularly night and morning, with the effect of completely preventing the sweating. The fever lasted fourteen days. In another case of acute articular rheumatism, atropia was given first in doses of one, then of two milligrammes, with a similar result ; and it is remarked that on two days in the course of the disease in which it was omitted, the sweating recurred. The atropia was given according to the following formula : Sulphate of atropia, six milligrammes (9-100ths of a grain;) extract of gentian, sufficient to make ten pills. Dr. Frantzel has never given larger doses than 1.2 milligrammes, (a little less than one-fiftieth of a grain,) from fear of producing toxic symptoms. Even doses of 0.6 and 1.2 milligrammes, though unattended with any mischief, have produced slight symptoms of poisoning. In not a few cases, after taking the medicine, the patient felt itching in the neck, which, however, disappeared in one or two hours. The pupils not unfrequently acted slowly, and were sometimes dilated. In some cases there were muscæ volitantes. The atropia had to be stopped in four cases on account of diarrhœa; that this was due to the medicine was proved by the fact that it ceased when the atropia was discontinued, and reappeared when it was resumed. What the physiological action of atropia is in arresting perspiration, Dr. Frantzel says it is difficult to determine. He is, however, inclined to believe that the profuse sweats arise from relaxation of the walls of the vessels supplied to the sudoriferous glands; and he remarks that the researches of Meuriot, Fleming, Jones, Hayden and Brown-Sequard have shown that atropia contracts the smallest vessels. To this are to be ascribed both the diminution of the sweats and the dryness of the mouth and skin observed in cases of poisoning with belladonna and with atropia.—*British Medical Journal, November* 29, 1873.

**Delirium Tremens.**

Dr. W. J. McMurray, of Nashville, Tennessee, in the *Medical and Surgical Reporter*, of February 14th, writes that in a severe case of delirium tremens, which resisted the usual remedies, he resorted to the injection of a strong decoction of coffee. He took one pint, in which he placed one ounce of castor oil, and gave the whole at one injection; this was at 3 A. M. From that time the patient never had another convulsion until 7 P. M., of the same day, when another injection of coffee was administered, which relieved him entirely of spasms. His pulse was reduced to infrequency, and he soon fell asleep and rested well during the night. He adds: "It seems as if the caffeine or some other property in the coffee has great control over the economy, by quieting the nervous system, equalizing the circulation, and thereby relaxing the muscles."

**Tape Worm.**

Dr. Chas. G. Bacon, of Fulton, N. Y., writes to the Phila. *Med. & Surg. Reporter*, extolling the use of pumpkin seeds in destroying tape worm. At 3 P. M., he gives pepo semina, (pulv.,) two ounces, in a pint of milk, instead of supper, patient having no dinner. At 9 P. M., same day, he administers the following:

℞ Olio ricini, ℥j; Olio terebinth, ʒij; Syrp. simplex, ℥ss. M. At 5 A. M., his patient passed the worm entire, 18 feet in length.

